# Oral disorders in children with Prader-Willi syndrome: a case control study

**DOI:** 10.1186/s13023-020-1326-8

**Published:** 2020-02-10

**Authors:** Carla Munné-Miralvés, Lluís Brunet-Llobet, Abel Cahuana-Cárdenas, Sergi Torné-Durán, Jaume Miranda-Rius, Alejandro Rivera-Baró

**Affiliations:** 1 Department of Pediatric Dentistry, Hospital Sant Joan de Déu, University of Barcelona, Barcelona, Spain; 2 Hospital Dentistry, Clinical Orthodontics and Periodontal Medicine Research Group (HDCORPEMrg), Institut de Recerca Sant Joan de Déu (IRSJD), Barcelona, Spain; 3 Department of Odontostomatology, Faculty of Medicine and Health Sciences, University of Barcelona, Barcelona, Spain

**Keywords:** Prader-Willi syndrome (PWS), Hyperphagia, Salivary alteration, Plaque index (PI), Caries index (CI)

## Abstract

**Introduction:**

Prader-Willi Syndrome (PWS) is a genetic disorder caused by the lack of expression of certain paternal genes located on chromosome 15q11-q13. This anomaly causes cognitive, neurological and endocrine abnormalities, among which one of the most important is hyperphagia. The aim of this study was to assess the oral health of children with PWA and to establish preventive criteria.

**Results:**

Thirty patients with PWS (mean age 10.2 years) and 30 age- and gender-matched controls were included in the study. Twenty-six patients with PWS(86.6%) followed dietary treatment prescribed by their endocrinologist. Individuals with PWS had a mean caries index of 53.3% and Decayed Missing Filled teeth (DMFT) index 2.5, and 53.3% had gingivitis, in the control group the respective figures were 43.3%, 0.93, and 60%. Only the DMFT index (*p 0.017*) presented significant differences. Regarding stimulated salivary secretion, patients with PWS presented a mean of 0.475 ml/min with a pH of 6.15, while controls presented a mean of 0.848 ml/min with a pH of 7.53; the differences between the groups were statistically significant in both cases (*p 0.032* and *p 0.0001* respectively). The population with PWS presented a higher plaque index (> 2) than their healthy peers, but the differences were not significant.

**Conclusion:**

Pediatric patients with Prader-Willi syndrome have an increased risk of caries and gingivitis. The children with this syndrome have a decreased salivary flow and a more acidic salivary pH. In these patients, dental care is an essential part of their multidisciplinary medical treatment.

## Introduction

The condition now known as Prader-Willi Syndrome (PWS) was first described by Langdon-Down in 1887, and the term Prader Labhart Willi Syndrome (later shortened to Prader-Willi Syndrome) was coined by Prader, Labhart and Willi in 1956 [1–5]. Currently, most authors agree that PWS is a complex multisystemic genetic disorder caused by the lack of expression of certain paternal genes located on the chromosome 15q11-q13 [4, 6, 7].

In 1981, Ledbetter demonstrated that most patients with PWS had an interstitial deletion of the proximal long arm of chromosome 15 at region q11–q13. Consequently PWS was described as one of the first examples of an error in genomic imprinting (GI) in humans. GI is a molecular process by which some genes are not expressed, due to the carrier parent. It is an epigenetic phenomenon in which the phenotype is modified depending on the mono-allele gene that is inherited from one of the parents. Therefore, the printing marks the genes in such a way that the origin of the two copies can be distinguished, with one parent’s copy being activated and the other’s copy silenced [8–10]. In the case of PWS, the process depends on the father, there is a fragment of the father’s chromosome 15q that is imprinted, that is to say, silenced, and so it is not expressed [9–11].

There are three genetic subtypes of PWS, whose common feature is the loss of expression of genes located at the 15q11-q13 locus. In descending order of frequency, these subtypes are: the deletion (DEL) of the 15q11-q13 region of paternal origin in about 65% of cases; maternal uniparental disomy (MUD) in about 30%, in which the individual presents two copies of the maternal chromosome 15; an imprinting center defect in less than 5% and rare cases of translocation involving the chromosome 15q11-q13 region [10]. This genetic anomaly leads to a pattern of physical characteristics with cognitive, neurological and endocrine abnormalities that change over the course of the patient’s life [12].

**Fig. 1:**
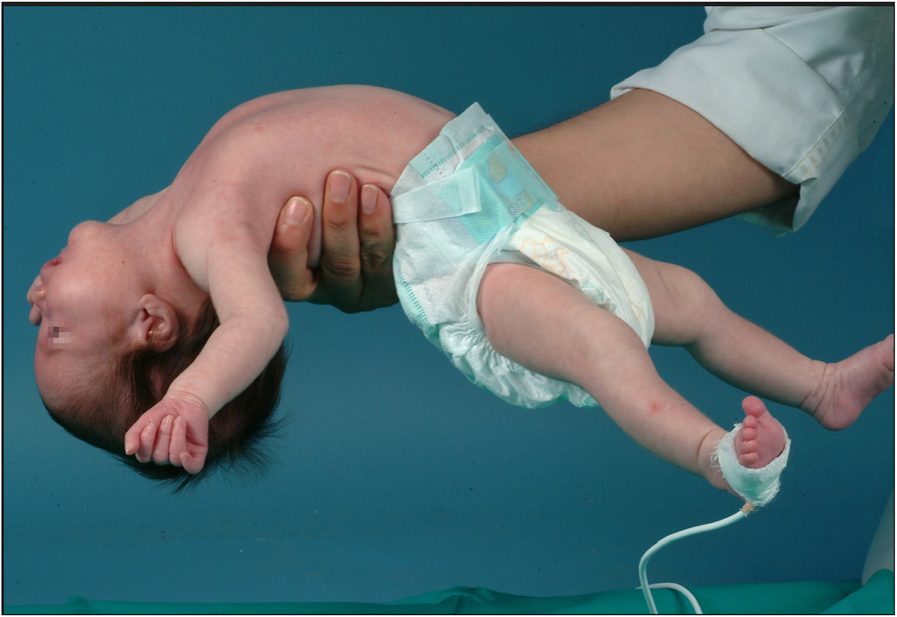
Hypotonia in a newborn with PWS

So PWS is a rare genetic disorder that can affect any race and sex [5, 8, 12–14] and has an estimated prevalence of 3.1: 100000 live newborns in Europe [15]. With regard to the patient’s eating habits, it is characterized by two stages. Newborns present severe hypotonia (Fig. 1), global developmental delay and lack of appetite. As a result they have difficulty gaining weight, and in most cases, feeding via nasogastric tube is necessary [4, 6, 13]. The second stage begins during early childhood; it is characterized by an eating disorder known as hyperphagia, which favors overweight and obesity [4, 6, 13, 16, 17].

In fact, PWS is the genetic syndrome most commonly associated with obesity, and its complications decrease life expectancy [4, 6, 9, 18, 19]. Apart from hyperphagia, patients also have a malfunctioning hypothalamus. The hypothalamus is a part of the brain that is responsible for integrating the nervous and endocrine systems which, together with the pituitary system, regulate aspects of growth, development, metabolism and homeostasis. Its main functions are the control of the autonomic nervous system by regulating visceral activity and the secretion of several glands; the production of hormones such as the growth hormone; the regulation of emotions, memory and behavior; the regulation of appetite; control of body temperature, and regulation of the heart rate and states of consciousness [20].

**Fig. 2:**
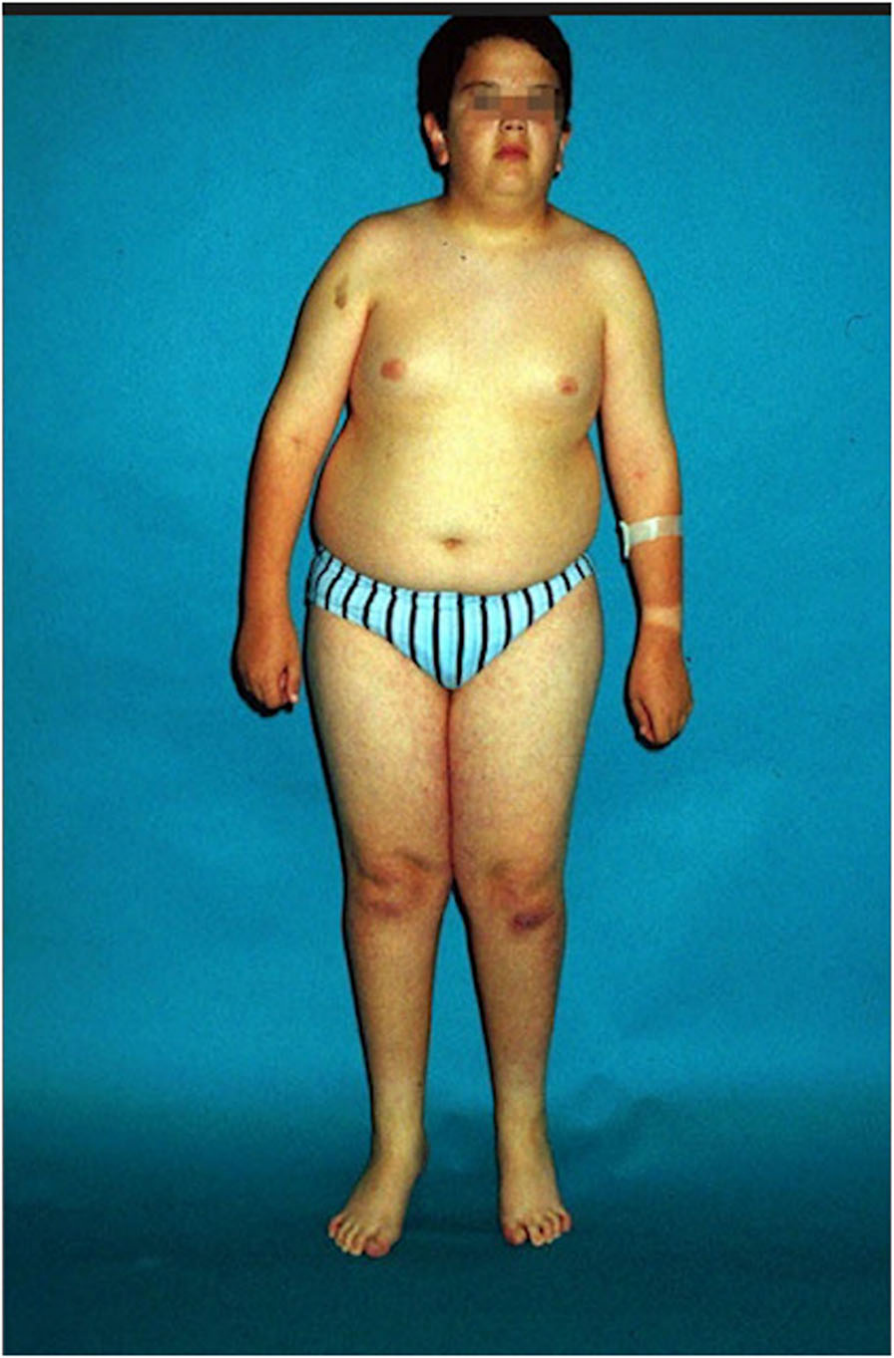
Physical characteristics of PWS

As a result, hypothalamic alterations can cause intellectual disability, behavioral problems, thermoregulatory dysfunction, a high pain threshold, respiratory sleep disorders, hypopigmentation, hypogonadism, pubertal delay and infertility, short stature, and small hands and feet (Fig. 2) [8, 21].

As for the facial phenotype, patients with PWS usually present a narrow forehead, elongated almond-shaped eyes with ascending oblique palpebral fissures, and a triangular mouth with commissures facing downwards and thin upper lip [1, 22–24].

**Fig. 3:**
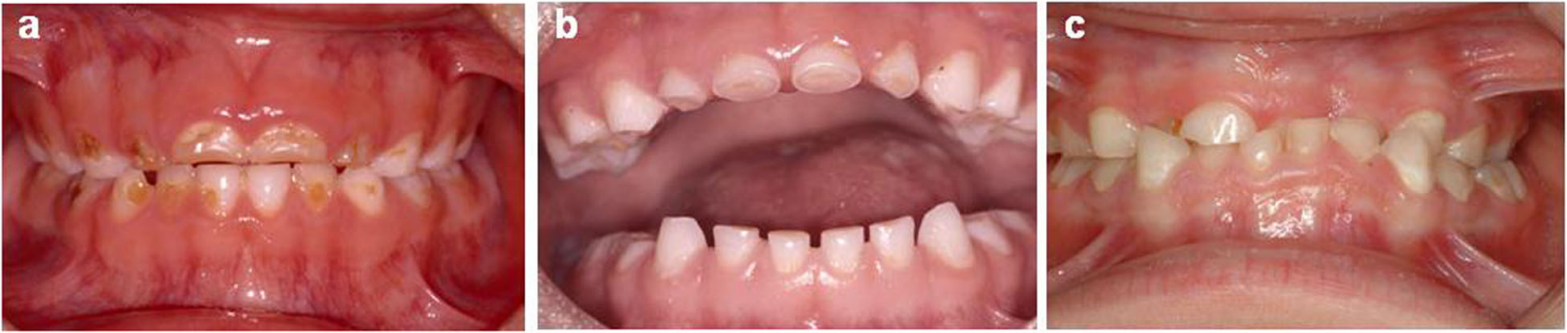
a.b/ Deciduous dentition. Observe the areas of attrition with severe incisal wear, and erosions present in vestibular areas; c/ Deciduous dentition. Dental malocclusion with anterior crossbite in the incisor region

The dietary problems associated with this general systemic condition increase the risk of oral pathology. It has been observed that patients with PWS are at greater risk of suffering oral diseases such as enamel hypoplasia, caries, tooth wear caused by attrition, erosion or abrasion, periodontal disease, delayed tooth eruption, candidiasis, oral lesions and decreased salivary flow (Fig. 3) [1, 18–20, 22, 24–26].

**Fig. 4:**
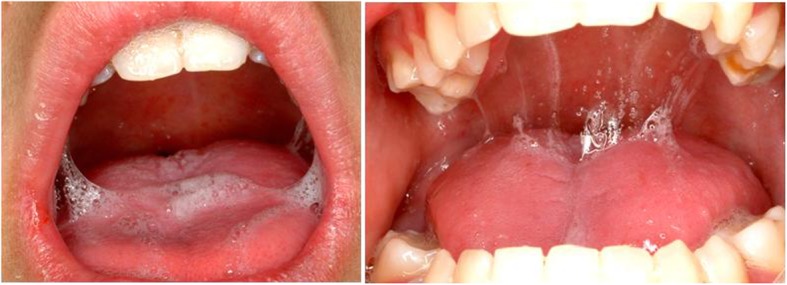
PWS patients present thickened, sticky saliva. See the clinical manifestations in the images

The Prader-Willi syndrome is characterized by low salivary quantity and quality due to atrophy of the salivary glands, which in turn is due to the low birth weight. Increased amounts of salivary ions and proteins make the saliva sticky, sparse and unable to perform its functions (Fig.4) [25, 27, 28]. The enamel hypoplasia may be caused by malnutrition and low birth weight, and is associated with the first phase of the syndrome [27, 29]. In addition, the combination of the lack of oral hygiene, reduced salivary flow and the preference for foods high in carbohydrates raises the risk of caries and gingivitis [18, 19, 24, 30]. These alterations in the oral cavity may be aggravated by the hypotonia which hinders suction, swallowing and chewing and makes the introduction of a soft diet mandatory [25].

The aim of this case-control study was to evaluate the oral conditions of children with Prader-Willi syndrome and to establish a prevention protocol for these patients.

## Materials and methods

A cross-sectional observational case-control study was designed and carried out between 2016 and 2017, in which 60 participants from the Sant Joan de Déu Hospital were enrolled: 30 patients with PWS (cases) and 30 age- and gender-matched healthy patients (controls).

Participants’ socio-demographic variables were recorded in a questionnaire. The nature of the study was explained to the parents or guardians who provided signed informed consent, prior to their children’s enrolment. If a child was following a medically prescribed diet, this was recorded. Both the oral examination and the salivary sampling were carried out in the dental office.

The type of dentition of each patient was registered and classified into deciduous, mixed or permanent. Subsequently, the Decayed Missing Filled teeth (DMFT) index was calculated for each individual and the accumulation of plaque was estimated using the Silness and Löe plaque index [31]: 0- No plaque in the gingival area; 1- A film of plaque adhering to the free gingival margin and adjacent area of the tooth. The plaque may be seen in situ only after application of disclosing solution or by using the probe on the tooth surface; 2- Moderate accumulation of soft deposits within the gingival pocket or the tooth and gingival margin, visible with the naked eye; and 3- Abundance of soft matter within the gingival pocket and/or on the tooth and gingival margin.

The presence or absence of gingival inflammation (gingivitis) was evaluated using the Löe and Silness gingival index [32]: 0- Normal gingiva, no inflammation, no change of color, no bleeding; 1- Mild inflammation, slight change in color, slight edema, does not bleed when probing; 2- Moderate inflammation, redness, edema and glazing, bleeding on probing and pressure; and 3- Severe inflammation, marked redness and edema, ulceration with tendency to spontaneous bleeding.

Finally, tests were carried out to quantify salivary pH. To obtain the saliva, we used the GC Saliva Check Buffer® Kit, a test specifically designed to assess saliva volume. Each patient was asked not to eat for two hours and to chew a paraffin tablet for two minutes and the volume of stimulated saliva was recorded, considering a value of 1.2 ml/min to be normal. Once this process was complete, saliva pH was measured by applying a drop of saliva on top of a Lyphan® test strip (Neuhaus, Germany). A score between 6.75 and 7.25 was considered normal.

After obtaining the results, the descriptive statistics of the study sample were recorded. The between-groups comparisons were made by means of the Chi-square test for qualitative variables and the Student’s t-test and Spearman’s rho for quantitative variables, accepting a p level < 0.05 as significant.

## Results

Of 43 patients genetically diagnosed with PWS at the Sant Joan de Déu Hospital, 30 were attended at the dentistry. All of them received growth hormone treatment. Both case and control populations consisted of 12 females and 18 males, with a mean age of 10.2 years (range 2–18 - SD 5.314). Twenty-six patients with PWS (86.6%) were receiving low calorie and low sugar dietary treatment in order to avoid obesity and its possible consequences.

Dentition was deciduous in 20% of the cases, mixed in 46.7% and permanent in 33.3%; in the control group the figures were 26.7, 40.0 and 33.3% respectively. Patients with PWS had a caries index of 53.3% and a DMFT of 2.5 (SD 3.170), and 16 (53.3%) had gingivitis. The controls had a caries index of 43.3% and a DMFT of 0.93 (SD 1.311), and 12 (40%) had gingivitis. About 70% of the population with PWS had a higher plaque index (grade > 2) than controls, of whom 56% presented mild plaque (grade < 1). PWS presented stronger associations with clinical gingivitis than controls, but the differences were not significant. Other findings identified during the bucodental examination in the PWS group were dental erosion, attrition or wear in nine (30%), angular cheilitis in three, and enamel hypoplasia in only two. In the group of healthy patients only three presented attrition or wear, and none presented angular cheilitis/oral lesions (Table 1).

**Table 1:** Descriptive statistics of the oral conditions in the sample

Parameters			PWS *n* (%)	Control *n* (%)
Sex	Male		18 (60%)	18 (60%)
	Female		12 (40%)	12 (40%)
Age	Mean age (years)		10.2 years (range 2 to18 - SD 5.314)	
Type of dentition	Deciduous	*n* (%)	6 (20.0%)	8 (26.7%)
		Mean age	3.16	3.87
	Mixed	*n* (%)	14 (46.7%)	12 (40.0%)
		Mean age	8.66	9.5
	Permanent	n (%)	10 (33.3%)	10 (33.3%)
		Mean age	16.2	16.1
Plaque Index		0	3 (10.0%)	2 (6.7%)
		1	6 (20.0%)	15 (50.0%)
		2	14 (46.7%)	8 (26.7%)
		3	7 (23.3%)	5 (16.6%)
Gingival Index		0–1	14 (46.7%)	18 (60.0%)
		2	16 (53.3%)	12 (40.0%)
		3	0	0
Dental erosion, attrition or wear			9 (30.0%)	3 (10.0%)
Enamel hypoplasia			2 (6.7%)	0
Angular cheilitis-oral lesions			4 (13.3%)	0
Candidiasis			0	0

In the tests to determine the quantity of stimulated saliva in ml/min and the pH, not all the participants were able to cooperate fully, due to intellectual disability in some cases and young age in others. Greater cooperation was obtained from healthy patients than from patients with PWS. Regarding stimulated saliva, the mean volume for cases was 0.475 (SD – 0.571) ml/min with a mean pH of 6.15 (SD – 0.818), compared with figures of 0.848 ml/min (SD – 0.493) and 7.53 (SD – 0.776) respectively in the controls (Table 2).

**Table 2:** Comparison of stimulated salivary flow, pH and DMFT in the two populations

	GROUP	n	Mean	SD	*P* value
ml/min	PWS	18	0.475	0.5714	0.032
	Control	25	0.848	0.4932	
pH	PWS	27	6.15	0.818	0.0001
	Control	30	7.53	0.776	
DMFT	PWS	30	2.5	3.170	0.017
	Control	30	0.93	1.311	

*p* < 0.05. Student *t*-test

The between-group comparison revealed statistically significant differences both in the DMFT index (*p 0.017*) and in the quantification of ml/min (*p 0.032*) and pH of the saliva (*p 0.0001*): PWS cases presented lower salivary flow, a more acidic pH and a higher DMFT index than controls (Table 2). Comparing the DMFT index versus stimulated saliva, saliva pH and the plaque index, the only statistically significant differences were observed in healthy individuals between the DMFT (0.93) and saliva pH (7.53). With more alkaline pH, the controls’ caries indices were lower (*p* < 0.05) (Table 3).

**Table 3:** Comparison of DMFT index and pH

DMFT and pH in healthy patients	Correlation coefficient	0.373*
	Significance (two-sided)	0.042
	*n*	30
DMFT and pH in patients with PWS	Correlation coefficient	−0.150
	Significance (two-sided)	0.455
	*n*	27

*p* < 0.05 (Spearman’s Rho test)

## Discussion

PWS is a genetic alteration that causes a hypothalamic dysfunction, which manifests itself in the form of physical, neurological and endocrine disorders, above all hyperphagia. Several hypotheses have been proposed regarding the eating disorders associated with the syndrome. McAllister et al. (2011) suggested that during the first years of life in these patients an excess of leptin, a satiating hormone, may favor poor feeding. At later ages, ghrelin, an appetite-regulating hormone, may be involved in the development of hyperphagia. PWS seems to be related more to the lack of satiety than to the feeling of hunger [17]. Because of their lack of control over feeding, patients must follow a strict fiber-rich diet (1100-1200Kcal) monitored by an endocrinologist [4, 6, 26].

PWS is a rare disease, most of the studies of the syndrome published to date are clinical case reports, and few studies have been carried out with large samples. At our institution we were able to group together a relatively large sample of patients under the “Prader Willi functional plan”, in which different services perform clinical controls of these patients at the same time and on the same day. For this reason, we were able to study a range of variables that provided us with a broad overview of the oral-dental health status of patients with PWS.

Due to the patients’ general state of health the risk of caries is high, even during the first year of life. Patients should be referred to the pediatric dentist, and periodontal prophylaxis should be performed every 3–4 months [33]. Several authors attribute the increased incidence of caries to poor oral hygiene and increased frequency of food consumption, in combination with alterations in saliva (in terms of quantity and viscosity) [18–20]. In an acidic environment, tooth enamel undergoes a process of demineralization which increases the risk of tooth decay. Saliva flow after food intake helps increase plaque pH, which has previously fallen due to the exposure to sugars and carbohydrates in the diet [34].

However, Saeves et al. (2012) found no significant difference between the risk of caries in individuals with PWS and in healthy patients [22]. In the present study, no significant differences were found regarding the caries indices between the populations, though caries were more frequent in individuals with PWS. In comparison to PWS patients, cerebral palsy patients of a similar age treated at our pediatric dentistry department presented lower scores on the DMFT index (2.8) and the plaque index (70% grade 3) and lower rates of gingivitis (80% moderate-severe).

According to Dougall and Fiske (2008) the poor oral hygiene of patients with PWS predisposes them to oral diseases [19]. Even so, recent research has reported an improvement of oral hygiene compared with earlier studies [4, 6]. With regard to periodontal disease, in a clinical case study of periodontitis in Prader-Willi Syndrome Yanagita M et al. (2011) concluded that periodontal loss was a consequence of the combination of dental malocclusion, occlusal trauma and poor plaque control. In addition, the interaction of multiple affected genes in PWS and a growth hormone deficiency may alter the immune response and increase susceptibility to periodontal destruction [24, 35]. The dental examination performed in the present study did not identify periodontitis in any of our patients, all of whom were treated with growth hormone.

Olczak-Kowalczyk D et al. (2019) showed that the mean gingival index values in PWS patients with mixed dentition, were significantly higher than in the control group, but the mean plaque index values were not [36]. In our sample there were more cases of gingivitis in patients with PWS than in their healthy peers, but the differences were not statistically significant.

Most authors agree that patients with PWS present reduced salivary flow. Saeves et al. (2012) suggest that the secretion of proteins in the salivary glands may be functioning well or may even be overexpressed, but that the reduction in flow affects both the quality and the amount of saliva [22, 23]. Although patient cooperation was a limitation for obtaining saliva, we found that the saliva of patients with PWS was more viscous and thick. Some researchers have reported that the type of saliva of these patients, with reduced flow rate and molecular and ionic changes, is incapable of remineralizing the enamel and, therefore, promotes excessive tooth wear and increases the risk of cavities [28, 37]. Olczak-Kowalczyk D et al. also confirmed worse physical and chemical parameters of saliva in subjects with PWS compared to the control group. They found a negative correlation between the age of PWS patients and watery consistency of saliva, and suggested that salivary viscosity may increase with age [36]. As we mentioned above, the difficulty of obtaining saliva in some non-collaborative patients meant that our study lacked the statistical power needed to carry out an in-depth analysis of this correlation between age and salivary viscosity. It has also been observed that inadequate salivary secretion may increase susceptibility to infections or lesions in the oral mucosa, and may increase the accumulation of dental plaque [20, 38]. Even so, a study of saliva and dental caries concluded that there was little association between values of salivary secretion and the incidence of caries [39]. Although the methods we used to obtain saliva were different from those used by González et al. (2008) [25] and Saeves et al. (2012) [23], our stimulated salivary secretion level was markedly lower than the value stipulated as normal (1.2 ml/min). In addition, our mean pH (6.15) was slightly more acid than normal (6.75–7.25) [40].

To be able to provide dental treatment for patients with PWS and to obtain good levels of oral hygiene, their ability to cooperate must be evaluated. A thorough clinical history must be obtained and the presence of oral diseases assessed [18]. To maintain adequate oral hygiene, Dougall and Fiske note the usefulness of drawings and posters with written text, because individuals with PWS have good visual ability [19]. The application of topical fluoride gels or daily rinses is recommended and frequent preventive dental appointments should be scheduled [19]. Other authors affirm that patients able to cooperate can undergo dental procedures under local anesthesia; due to patients’ poor muscle control, they recommend the use of mouth openers [18]. Several researchers stress the importance of an early diagnosis, the observation of basic prevention guidelines from the first year of life, and a multidisciplinary approach to the disease so as to improve quality of life [4, 6, 8, 10, 30, 33].

Within the multidisciplinary team, the role of the pediatric dentist is essential for the management and establishment of oral prevention guidelines for children with PWS. It is important to coordinate the appointments with different specialists on a single day, so as not to disrupt families’ daily routines and to avoid multiple hospital visits. The protocol described in this study was designed by a committee of experts from the same hospital and was led by the endocrinology team. Other services such as genetics, neurology, traumatology, ophthalmology psychology, gastroenterology, pulmonology and dentistry, coordinated periodical visits every 6–12 months.

## Conclusions

Pediatric patients with Prader-Willi syndrome have a higher risk of cavities and gingivitis than the general population. Children with this syndrome have decreased salivary flow and a more acid salivary pH.

Although several hypotheses have been put forward regarding the eating disorders associated with this syndrome, it is important to continue the study of the processes that hinder the control of hyperphagia and the impact of this eating disorder on oral health.

Finally, Prader-Willi syndrome is associated with an increased risk of certain oral-dental diseases in pediatric ages. Establishing a prevention protocol that enables pediatric dentists to provide dental care at very early stages of life is an essential component of the multidisciplinary therapeutic approach in these patients.

## Data Availability

The datasets used and/or analysed during the current study are available from the corresponding author on request.

## Abbreviations

DMFT: Decayed Missing Filled teeth index
MUD: Maternal uniparental disomy
PWS: Prader-Willi Syndrome
